# Clinical significance of family accommodation and parental psychological distress in a sample of children and adolescents with obsessive-compulsive disorder aged 8-17 years old

**DOI:** 10.1186/s13052-020-00932-2

**Published:** 2020-11-10

**Authors:** Maria Pontillo, Francesco Demaria, Maria Cristina Tata, Roberto Averna, Prisca Gargiullo, Maria Laura Pucciarini, Ornella Santonastaso, Tommaso Boldrini, Alberto Eugenio Tozzi, Stefano Vicari

**Affiliations:** 1 Department of Neuroscience, Child and Adolescent Neuropsychiatry Unit, Children Hospital Bambino Gesù, Viale Ferdinando Baldelli, 41, 00146 Rome, Italy; 2 Department of Dynamic and Clinic Psychology, Faculty of Medicine and Psychology, Sapienza University of Rome, Via dei Marsi, 78, 00185 Rome, Italy; 3 Multifactorial Disease and Complex Phenotype Research Area, Bambino Gesù Children’s Hospital, IRCCS, Rome, Italy; 4 Institute of Psychiatry, Fondazione Policlinico Universitario A. Gemelli, Catholic University, 00168 Rome, Italy

**Keywords:** Family Accommodation, Obsessive-Compulsive Disorder, Functioning, Children, Adolescents, Parental psychological distress

## Abstract

**Background:**

Family Accommodation (FA) refers to the involvement of family members (especially parents) in the compulsive behaviors of children and adolescents with Obsessive-Compulsive Disorder (e.g. modifying family routines or facilitating avoidance of obsessive-compulsive triggers). Many studies have examined the high prevalence of FA in this clinical population; however, less is known about its clinical significance and relationship to the individual psychological distress of parents. In our study, we investigated the clinical significance of FA examining its relationship with obsessive-compulsive symptomatology, functioning, anxiety and depressive symptoms in a clinical sample (*n* = 51) of children and adolescents with Obsessive-Compulsive Disorder (OCD) aged 8–17 years old and their parents, included to examine their individual psychological distress.

**Methods:**

The sample was divided into two groups: the High Accommodation group (*n* = 36) and the Low Accommodation group (*n* = 15).

**Results:**

Results demonstrated that children and adolescents in the OCD High Accommodation group reported major functional impairment in global (*p* = .001313), social (*p* = .000334) and role (*p* = .000334) domains, and higher depressive symptoms than the Low Accommodation group. Both fathers and mothers from the High Accommodation group reported a higher level of individual psychological distress compared to mothers and fathers from the Low Accommodation group (*p* = .040365).

**Conclusions:**

The findings of this study show that FA is common in children and adolescents with OCD and it could cause not only an impairment of the patient’s global, social and role functioning but also a high level of individual psychological distress in the single parent. The presence of FA should therefore be carefully investigated and considered in planning assessment and treatment of OCD in children and adolescents.

## Background

Obsessive–Compulsive Disorder (OCD) is a neuropsychiatric disorder characterized by obsessive thoughts, meaning intrusive, repetitive, and unwanted thoughts, associated with compulsive behaviors [1]. OCD is related to a significant impairment in quality of life and pediatric OCD leads to a deterioration of functioning in social, scholastic, and family contexts [2–5]. OCD occurs with worldwide prevalence rates ranging 0.25–3.0% [6] and presented high comorbidity rates with other psychiatric disorders [7]. Moreover, in about 50% of adult OCD cases, patients report that their obsessive-compulsive symptomatology started before 18 years [8].

In patients with OCD, compulsive behaviors could implicate the involvement of a family member that accommodates the pathology (e.g. provide objects needed for rituals). Moreover, family members can accommodate compulsive symptoms by performing rituals for the patients (e.g. checking, cleaning), modifying family routines, providing reassurance, or facilitating avoidance of OCD triggers. These modifications are implemented with positive intentions, to decrease patient’s distress and time occupied executing compulsions [9]. More than 90% of parents report at least some accommodations [10, 11].

Studies conducted on children and adolescents with OCD [12] and adults with OCD [12–15] have shown that the presence of Family Accommodation (FA) is associated with a major level of severity of the OCD symptomatology with a consequence of maintaining and exacerbating OCD symptoms and increasing the request for help during rituals [16]. Indeed, although family members engage in these behaviors to attenuate OCD-related distress and diminish the time occupied by symptoms, they reinforce the belief that is important to respond to OCD implicit thoughts. In this way, patients continue acting on OCD-related compulsions but, due to Family Accommodation, they do not recognize a significant decrease in functioning, experiencing less distress and impairment [17, 18]. At the same time, general family functioning decreases, while increasing family members’ distress, resulting in high levels of family conflict, and major expressed emotions, defined as criticism, hostility and emotional over-involvement [4, 19–23]. To further support this, Family Accommodation correlated negatively with family functioning and positively with family stress [11].

Interestingly, the type of parental relationship between patients with OCD and those who perform accommodation behaviors does not influence this correlation that is the same between parent-child, spouses, or siblings [16].

Several studies [24–26] proposed that Family Accommodation is ubiquitous in children and adolescents with OCD. Indeed, children have a relationship with the family in a different manner than adults [27]. For example, children and adolescents depend on their family for guidance in most domains of daily life and spend a lot of time with their parents helping them with many tasks. Thus, families have considerable opportunity to maintain children’s obsessive-compulsive symptoms [28].

Confirming this, Storch et al. [26] reported a high prevalence of Family Accommodation in a sample of 57 children and adolescents (mean age: 12.99 ± 2.54 years) and their parents. In particular, high prevalence of Family Accommodation was associated with a major severity of OCD symptomatology and a consequent major functional impairment of the children and adolescents, primarily in family functioning, compared to social and academic functioning.

In a recent study, Wu et al. [9] investigated the clinical profiles of Family Accommodation in 150 pediatric OCD subjects (mean age: 12.39 ± 3.07 years). The entire sample (100%) reported the presence of Family Accommodation. In particular, 80% of the sample refer to providing reassurance to the child, while 70.7% refrain from saying or doing things in consequence of OCD symptoms. Moreover, in line with the studies described above, Family Accommodation correlated positively with OCD symptom severity, according to Children’s Yale-Brown Obsessive–Compulsive Scale (CY-BOCS) [29].

About that, Wu et al. [9] explain that the sample presented a high level of OCD symptom severity, with high frequency of cleaning and contamination symptoms, and low general functioning.

These results replicate those of a precedent meta-analysis of Wu et al. [30], based on 41 studies, that reported a linear correlation between the severity of OCD symptoms and a major presence of Family Accommodation, without a significant correlation with age, comorbid disorders and types of tools used to assessment [30]. Overall, these three studies are the only studies in which the role of Family Accommodation is studied in a clinical sample consisting only of children and adolescents. Other studies, in fact, provide a wider age range [16, 30–32]. Also, although these studies showed a positive correlation between Family Accommodation and the severity of OCD symptoms, their cross-sectional design limits the possibility to draw inferences about the causality of this relationship. Even if it is unclear which direction of causality is present, the authors affirm that this relationship is likely bidirectional. In fact, a major presence of OCD symptoms could results in a major performance of accommodating behavior in order to mitigate distress and facilitate functioning; conversely, Family Accommodation could contribute to maintaining OCD symptomatology and correlated anxiety [9, 30] preventing the child from experiencing habituation of anxiety and learning that feared consequences typically do not occur. However, these results are not sufficiently supported.

In addition, in literature, there are few studies that systematically investigated the relationship between Family Accommodation and individual psychological distress in family members of OCD patients.

In fact, participating in a patient’s compulsions, providing reassurances and helping to avoid anxiety-inducing stimuli, imply a modification of family routine and a deterioration of parental quality of life, with consequent feelings of depression, guilt, anger, frustration and shame [11, 33–35].

In particular, the first study investigated the consequences of Family Accommodation on caregivers was proposed by Calvocoressi and colleagues [11], based on 34 participants (20 spouses and 14 parents) of 34 young adults and adults with OCD (mean age: 35.2 ± 11.4 years; range age: 20–75). Results show a significant presence of relative distress associated with a severe presence of Family Accommodation, with extreme modifications of relative’s functioning [11].

Also, Cosentino et al. [36], showed that, in a sample of 31 relatives of 19 OCD patients (mean age: 27.79 ± 8.28; range age: 15–55), family members inclined to accommodation reported a major presence of guilt sensitivity, anxiety sensitivity, and a passive communication style [36]. However, both of these studies were conducted on family members of OCD adults and not on family members of OCD children and adolescents.

### Aims

Based on the literature described, the aims of our study were: 1) to examine in detail the clinical significance of Family Accommodation and its relationship to obsessive-compulsive symptomatology, functioning, and anxiety and depressive symptoms in a clinical sample of children and adolescents with OCD aged 8–17 years old. The possible effect of pharmacological or psychological treatment on these relationships was taken into consideration.

2) To examine the possible differences in the level of individual psychological distress of parents that accomplish high Family Accommodation in children and adolescents with OCD disorders compared to parents that accomplish low Family Accommodation.

## Methods

### Participants

Participants in this study were 158 children and adolescents aged 8–17 years consecutively admitted to the Child and Adolescent Neuropsychiatry Unit of the Clinical and Research Hospital Bambino Gesù of Rome for obsessive-compulsive symptoms between January 2018 and August 2019. All participants were drug-naïve patients at the time of clinical assessment and did not receive psychosocial interventions. We included children and adolescents with pediatric OCD who did not require hospitalization. All participants and their parents/legal guardians provided written informed assent and consent. The study was approved by the Ethics Committee of the Children’s Hospital.

### Procedures

#### Clinical assessment of children and adolescents

All participants (*n* = 158) were assessed with the Schedule for Affective Disorders and Schizophrenia for School Aged Children Present and Lifetime Version DSM -5 (K-SADS-PL DSM-5) [37], a semi-structured interview that assesses the presence of mental disorders according to DSM-5 classification [1]. Neurocognitive functioning (IQ) was measured with the Wechsler Intelligence Scale for Children (WISC-IV) [38].

The inclusion criteria for this study were presence of OCD as a primary diagnosis based on DSM-5 [1] and IQ > 70. Exclusion criteria was the presence of OCD symptoms in patients with a primary diagnosis of Autism Spectrum Disorder, Attention Deficit/Hyperactivity Disorder, Tourette Disorder and Tic Disorder. Of whole sample (*N* = 158), 51 subjects (32.3%) met the inclusion criteria, received a primary diagnosis of OCD and were included in the study, the remaining 107 (68%) were excluded for previously defined exclusion criteria (e.g. obsessive-compulsive symptoms in Tourette disorder or in Intellectual Disability).

The final sample composed of 51 subjects with OCD was assessed with the Children’s Yale–Brown Obsessive Compulsive Scale (CY-BOCS) [29], a clinician-rated semi-structured interview that assesses the presence and severity of obsessive and compulsive symptomatology. According to the literature [39], the CY-BOCS is the gold-standard instrument for the evaluation of OCD symptomatology.

All 51 subjects were also assessed for level of functioning and presence of depressive and anxiety symptoms associated.

The level of functioning was measured with the Childhood Global Assessment Scale (CGAS) [40]. Furthermore, social and role functioning was specifically assessed with the Global Functioning: Social Scale (GF: Social) [41] and the Global Functioning: Role Scale (GF: Role) [42] to obtain differential measures of functioning.

Depressive symptoms was assessed using The Child Depression Inventory 2 (CDI 2) [43], a self-report questionnaire. Two scales formed the total score: the emotional problem scale and the functional problems scale. The emotional problem scale is composed of two subscales: the negative mood/physical symptoms scale which examined the presence of depressive mood and neurovegetative symptoms; and the negative self-esteem scale, which examined the negative self-perception of the patients. The functional problems scale is also composed of two subscales: the ineffectiveness scale, which examined the functional general problems due to depressive symptoms; and the interpersonal problems scale, which investigated the interpersonal expression of depressive mood.

Anxiety symptoms were measured using the Multidimensional Anxiety Scale for Children 2 (MASC 2) [44], a self-report questionnaire. The total score of the MASC 2 is formed by six scales: the separation anxiety/phobias index; the general anxiety disorder index; the social anxiety index, composed of the humiliation/rejection subscale and the performance fears subscale; the obsession and compulsion index; physical symptoms, composed of the panic subscale and the tense/restless subscale; and the harm avoidance index.

#### Clinical assessment of parents

Parents of 51 subjects with primary diagnosis of OCD separately completed the Family Accommodation Scale (FAS) [18], a self-report questionnaire that measures the degree of the family members’ involvement in the patient’s obsessive-compulsive symptomatology. The questionnaire is composed of 13 items: items 1–9 provided a total Family Accommodation score and, in particular, investigated the relatives’ involvement in the patient’s OCD symptoms (items 1–5) and the functional impairment due to the involvement in symptoms (items 6–9); item 10 indicates the consequence level of stress experienced by parents; items 11–13 investigated the consequences of not complying with the patient’s symptoms.

To measure the parent’s psychological distress, each parent completed the Symptom Checklist-90-R (SCL-90-R) [45], a self-report checklist that examined the internalization and externalization of symptoms. SCL-90-R was composed of nine principal symptomatologic dimensions: Somatization (disease linked to bodily disrespect); Obsessive-Compulsive symptoms; Interpersonal Sensitivity (feelings of inadequacy and inferiority); Depression; Anxiety; Hostility; Phobic Anxiety; Paranoid Ideation; Psychoticism (interpersonal alienation). The SCL-90-R is additionally composed of three global indexes: Global Severity Index (GSI), a summary index based on the number of reported symptoms and the intensity of experienced discomfort; Positive Symptom Distress Index (PSDI), which examined the accentuation or minimization of responses; Positive Symptom Total (PST), a measure of the number of reported symptoms.

### Statistical analysis

Data were analyzed using SPSS IBM Statistics version 20 statistical software (IBM Corp, Armonk, NY, USA). First, we calculate the prevalence of FA in parents of all 51 subjects with OCD. We then divided the 51 subjects into two groups based on the mean score of mothers and fathers in the involvement scale (item 1–9) on the FAS. The first group was composed of children and adolescents with OCD whose parents had an item 1–9 FAS mean score of < 10 (Low Accommodation group; *n* = 15). Children and adolescents with OCD whose parents had an item 1–9 FAS mean score of ≥10 composed the second group (High Accommodation group; *n* = 36). For the composition of the two groups, we chose the FAS cut-off score based on literature data [11, 36]. Indeed, the validation study of the FAS identifies the score of 10 as the starting point of moderate gravity range for presence of Family Accommodation. The two groups were unequal in size, but Levene’s test confirmed homogeneity of variance and the Shapiro–Wilk test confirmed the normal distribution of the variables based on continuous data. Separate group comparisons based on one-way ANOVA were performed on demographic and psychiatric variables, whereas the Chi Square Test was performed on frequency data.

## Results

### Sample characteristics

The final sample consisted of 51 subjects (mean age: 13.5 ± 2.7 years) with a diagnosis of OCD. Family Accommodation was reported by 100% of parents of these 51 subjects with OCD. Indeed, all parents, both mothers and fathers, scored above zero in at least one of the 1–9 items of the FAS.

Fifty-one subjects with OCD were divided into two groups, based on FAS total score, as explained in the paragraph above. The first group (Low Accommodation group) was composed of 15 children and adolescents with OCD, with a mean age of 13.8 ± 2.9 years. In this group, 66.7% of the subjects were males. The second group (High Accommodation group) was composed of *36* children and adolescents with OCD. This sample presented a mean age of 13.4 ± 2.5 years and 75% of the subjects were males. Parents of both groups reported reassurance and helping in avoidance as the most frequent types of Family Accommodation.

Finally, at the time of evaluation, no participant was subject to psychosocial or pharmacological treatment.

### Comparison between the two groups (High Accommodation group vs Low Accommodation group)

As shown in Table 1, there were no significant group differences for age (*F* (1,95) = 0.04, *p* = 0.837), IQ (*F* (1,33) = 0.46, *p* = 0.503), family unit (*F* (1,48) = 3.58, *p* = 0.646) or number of psychiatric diagnoses associated with OCD (*F* (1,49) = 0.88, *p* = 0.351).

**Table 1:** Socio-demographic data and psychiatric assessment scores separated by the two groups (Low vs High Accommodation)

Variable	Low Accommodation	High Accommodation	***p***-value
	*N* = 15	*N* = 36	
	Mean (sd)	Mean (sd)	
Age, years	13.8 (± 2.9)	13.2 (± 2.6)	.837337
IQ	106.2 (± 12.9)	101.5 (± 19.0)	.503295
Family unit	4.1 (± 0.8)	3.6 (± 0.9)	3.57645
Item 1–9: total Family Accommodation score	6.1 (± 0.3)	16.8 (± 0.55)	<.00001**
Number of psychiatric diagnoses	0.8 (± 0.7)	0.97 (± 0.56)	.351231
C-GAS	54.2 (± 1.6)	50.8 (± 3.5)	.001313*
GF: Role	4.5 (± 0.5)	4.0 (± 0.3)	.000334*
GF: Social	4.5 (± 0.5)	4.0 (± 0.3)	.000334*
CY-BOCS: Total	17.4 (± 9.2)	21.9 (± 9.3)	.121465
CY-BOCS: Obsessions	9.1 (± 4.6)	11.4 (± 4.9)	.134576
CY-BOCS: Compulsions	8.7 (± 4.4)	10.5 (± 4.9)	.121465
MASC – 2: Total	55.6 (± 12.9)	60.4 (± 14.2)	.315124
MASC – 2: Obsessions/Compulsions	52.8 (± 9.7)	59.3 (± 13.4)	.13182
CDI – 2: Total	49.5 (± 9.6)	57.5 (± 12.8)	.057318
CDI – 2: Negative self-esteem	49 (± 8.4)	54.7 (± 12.9)	.035004*
CDI – 2: Ineffectiveness	46.9 (± 6.8)	56.4 (± 12.8)	.017609*

*C-GAS* Children’s Global Assessment Scale, *GF: Social* Global Functioning: Social Scale, *GF: Role* Global Functioning: Role Scale, *CY-BOCS* Children’s Yale–Brown Obsessive–Compulsive Scale, *FAS* Family Accommodation Scale, *MASC-2* Multidimensional Anxiety Scale for Children-2, *CDI-2* Child Depression Inventory-2

*p < .05

**p < .0001

Concerning comorbidities, statistical analysis confirmed no differences between the groups (*χ2 =* 6.6623; *p* = .154848).

Regarding OCD symptomatology, there were no significant differences between the two groups in the type of obsessions and compulsions, according to CY-BOCS (Obsessions: χ2 = 4.4781; *p* = .345149; Compulsions: χ2 = 2.7755; *p* = .596063). Analysing the percentage frequency, in the Low Accommodation group there was a prevalence of miscellaneous obsessions (e.g. fear of doing something embarrassing, the need to know or remember things, fear of saying certain things) (46.7%) and contamination obsessions (33.3%), whereas in the High Accommodation group, contamination obsession was the most prevalent (55.6%). Regarding compulsions results, the Low Accommodation group presented higher frequency of checking compulsions (53.3%) compared to High Accommodation group, whereas the High Accommodation group presented higher frequency of washing/cleaning compulsions (38.9%).

#### Global, social and role functioning

Significant differences between the two groups were found in functioning. In fact, the High Accommodation group presented worse global functioning than the Low Accommodation group (*F* (1,45) = 11.75, *p* = 0.001); similarly, social and role functioning was lower in the High Accommodation group (social functioning: *F* (1,45) = 15.08, *p* = 0.0003; role functioning: *F* (1,45) = 15.08, *p* = 0.0003) compared to the Low Accommodation group.

#### Level of anxiety and level of depressive symptoms

The High Accommodation group also presented higher scores for depressive symptomatology, negative self-esteem (*F* (1,36) = 4.80, *p* = 0.035) and ineffectiveness (*F* (1,36) = 6.19, *p* = 0.018) on the CDI-2 subscale than the Low Accommodation group. The two groups were comparable for levels of anxiety symptoms (*F* (1,37) = 1.04, *p* = 0.315).

#### Parental psychological distress

Psychological profiles of parents involved in the study were investigated through the SCL-90-R, scored separately for mothers and fathers, as shown in Table 2.

**Table 2:** Differences between two groups, separately for mothers and fathers, in SCL-90-R

SCL-90-R	Low Accommodation	High Accommodation	***p***-value
	*N* = 15	*N* = 36	
	Mean (sd)	Mean (sd)	
**Fathers**			
Somatization	43.47 (± 9.16)	50.68 (± 7.86)	.010119*
Obsessive-compulsive	44.32 (± 8.87)	50.00 (± 6.17)	.021303*
Interpersonal sensitivity	48.42 (± 10.67)	48.63 (± 6.55)	.495909
Depression	46.84 (± 9.46)	47.89 (± 5.46)	.348621
Anxiety	49.84 (± 12.38)	50.58 (± 8.92)	.441336
Hostility	45.32 (± 9.34)	45.79 (± 4.08)	.442168
Phobic anxiety	47.16 (± 9.11)	47.53 (± 4.54)	.457973
Paranoid ideation	44.95 (± 10.08)	45.42 (± 5.48)	.438055
Psychoticism	44.42 (± 5.32)	47.63 (± 5.83)	.030716*
GSI: global severity index	43.53 (± 8.47)	48.58 (± 6.47)	.027020*
PSDI: positive symptom distress index	46.37 (± 10.93)	46.00 (± 9.40)	.418759
PST: positive symptom total	45.00 (± 9.30)	48.68 (± 6.35)	.086933
**Mothers**			
Somatization	47.21 (± 9.57)	50.26 (± 10.83)	.202046
Obsessive-compulsive	50.26 (± 11.91)	52.89 (± 12.99)	.287710
Interpersonal sensitivity	51.26 (± 11.94)	52.47 (± 11.70)	.407201
Depression	51.58 (± 11.91)	54.53 (± 9.37)	.201033
Anxiety	50.53 (± 10.74)	55.05 (± 12.18)	.133027
Hostility	48.32 (± 10.27)	52.47 (± 11.76)	.130722
Phobic anxiety	49.63 (± 8.08)	52.32 (± 11.53)	.239653
Paranoid ideation	47.84 (± 10.97)	51.47 (± 11.55)	.196747
Psychoticism	50.16 (± 9.26)	53.00 (± 11.15)	.201399
GSI: global severity index	49.37 (± 12.02)	56.47 (± 10.66)	.040365*
PSDI: positive symptom distress index	50.58 (± 13.34)	52.95 (± 11.19)	.332677
PST: positive symptom total	47.32 (± 11.73)	51.42 (± 10.73)	.134763

*p < .05

Regarding fathers, the GSI score presented significant differences between the two groups (*p* = .027020). Fathers from the High Accommodation group reported higher scores in the Global Severity Index (mean score: 48.58 ± 6.47) compared to the Low Accommodation group (mean score: 43.53 ± 8.47). Moreover, significant differences between the two groups were also found in the somatization scale (*p* = .010119) and obsessive-compulsive scale (*p* = .021303): fathers from the High Accommodation group reported higher scores in both of these scales. In addition, the GSI scores of the mothers presented significant differences between the two groups (*p* = .040365). Mothers from the High Accommodation group reported higher scores in the Global Severity Index (mean score: 56.47 ± 10.66) compared to the Low Accommodation group (mean score: 49.37 ± 12.02). There were no significant differences in other scales.

## Discussion

The main aim of the present study was to explore in detail the role of Family Accommodation in the clinical picture of a sample of children and adolescents with a primary diagnosis of OCD. The first result we obtained was that Family Accommodation is common in pediatric OCD: in our sample, the Family Accommodation carried out by the parents is present in the clinical picture of all (100%) the children and adolescents with OCD examined; the type of Family Accommodation with higher frequency was to provide reassurances. This is consistent with previous studies [25, 26] showing that Family Accommodation is ubiquitous in children and adolescents with pediatric OCD. For example, very recently Wu et al. [9] reported that, in a sample of 150 youths with OCD, 99.3% of parents showed some type of Family Accommodation (e.g. providing reassurance, refraining from saying/doing things).

By dividing our entire sample of children and adolescents with pediatric OCD into two groups based on the level of accommodation carried out by the parents (Low Accommodation vs High Accommodation), we found that the High Accommodation group demonstrated significantly poorer global functioning than the Low Accommodation group. Previous studies [9, 26] have shown that the presence of Family Accommodation was significantly related to a reduction of global functional impairment. Unlike Storch et al. [26] and Wu et al. [9], we also investigated the influence of FA on two specific functioning domains, role and social, in order to clarify the effect of Family Accommodation on the functioning of children and adolescents with pediatric OCD. We found that both of these aspects were more compromised in High Accommodation group than in Low Accommodation group. Interestingly, in our study, two groups (Low Accommodation vs High Accommodation) were not significantly different for levels of cognitive functioning, levels of anxiety or numbers of psychiatric diagnoses associated with OCD. In other words, in our group of patients with high FA, the presence of high FA carried out by parents could determine the poorer global, social and role functioning regardless of cognitive functioning, level of anxiety and comorbid psychiatric diagnoses. In addition, our results showed that our two groups of children and adolescents with pediatric OCD did not differ significantly in terms of degrees of severity of OC symptomatology (measured by CY-BOCS). Therefore, with the same severity of the OCD symptoms, the level of Family Accommodation carried out by parents could influence the level of global, social and role impairment associated with the OCD disorder. Based on these results, our proposal is that, in children and adolescents with obsessive-compulsive disorders of equal severity, the presence of high Family Accommodation could be associated with a greater impairment of not only global (which includes family) but also role (school) and social functioning. This could be explained by the fact that, if left untreated, Family Accommodation can cause children and adolescents with OCD to be more likely to engage not only in compulsive behaviours, but also in avoidance. Just the avoidance of the threat (e.g. contamination) could extend not only to the family context but also to the social and scholastic context thus making the functional impairment associated with the OCD wider and, therefore, more difficult to treat.

Interestingly, our findings showed that the High Accommodation group showed higher depressive symptoms, like negative self-esteem and ineffectiveness, compared to the Low Accommodation group. Therefore, the level of FA would affect the level of depressive symptoms regardless of other variables such as cognitive functioning, level of anxiety, comorbid psychiatric diagnoses, and severity of the OCD symptoms. Indeed, because of the high Family Accommodation and the consequent avoidance, children and adolescents with OCD disorders are prevented from developing appropriate behaviors to cope with their OCD-related distress. In other words, they cannot perceive themselves as being able to cope with their OCD symptoms and the associated threats; this could lead to a tendency to experience depressive symptoms with a negative self-image. Our considerations are in line with Wu et al. [9] who proposed that Family Accommodation operates contrary to the goals of exposure and response prevention, the first line treatment for OCD [46, 47]. Indeed, as suggested by Blakey et al. [48], within the Cognitive Behavioral Therapy (CBT) approach, exposure consists in repeated and prolonged confrontation with situations and stimuli that trigger obsessions (e.g., books ordered the “wrong way”). Response prevention includes resisting urges to perform behaviours such as avoidance and compulsive rituals (e.g., ordering) during and after exposure trials. Based on our findings, we propose that a specific treatment based on the reduction of Family Accommodation is necessary to enhance the compliance of children and adolescents with OCD to CBT exposure and the response prevention paradigm and to reduce functional impairment [49]. Specifically, family members should be driven to become aware of the negative implications of Family Accommodation on the maintenance of obsessive-compulsive symptoms and on the interference with the cognitive-behavioral treatment focused on the exposure and response prevention. In our study, we also examined the effect of FA on the level of psychological distress of the single parent. By carrying out separate analyses for mothers and fathers, we found that both mothers and fathers from the High Accommodation group reported higher scores in the Global Severity Index of SCL-90–R compared to mothers or fathers of the Low Accommodation group. The Global Severity Index of SCL-90–R is a measure of overall psychological distress of individuals in a given time frame (the last 7 days) thus providing information on the current psychological status of parents during the period of illness of the children or adolescents. In an interesting way, in our study, the parents who most assist the children in the implementation of compulsive rituals (High Accommodation group) have a higher level of individual psychological distress. We therefore propose that the FA could cause not only an impairment of the functioning of the children and adolescents with OCD and impairment of family functioning, but also bring about a high level of individual psychological distress in the single parent. This finding illustrates the need to build intervention proposals within the CBT approach that focus specifically on the FA and consider the associated level of individual psychological distress of the parents. Indeed, as shown by Iniesta-Sepulveda et al. [50], cognitive-behavioral family-based treatment (CBFT) offers a limited effect on Family Accommodation and, consequently, on individual psychological distress of the parents.

### Strengths and limitations

The strength of this study is to have examined in detail the relationship between the level of Family Accommodation and different elements (e.g. severity of obsessive-compulsive symptoms, level of global, social and role functioning, level of anxiety, level of depressive symptoms, number of psychiatric diagnoses associated with OCD) of the clinical picture of children and adolescents with OCD. Specifically, we evaluated the presence of OCD disorders in our sample using ‘gold standard’ instruments for the assessment of psychiatric disorders. We used K-SADS-PL and CY-BOCS that are semi-structured-interviews with levels of reliability and validity superior to those of self-report questionnaires. Additionally, we examined the level of global, role and social functioning using tools that require the clinician’s judgment and not with questionnaires filled out directly by parents or children or adolescents. The use of tools rated by experienced clinicians allows for a more reliable assessment of the functional impairment associated with the OCD; indeed, if the evaluation is performed directly by the child or adolescent, their experience of functional impairment may decrease as the degree to which increases the Family Accommodation performed by parents. For example, as Storch et al. [26] suggest, if the family facilitates tasks to minimize the child’s OCD-related distress, the child may not report a significant decrease in functioning. Likewise, if the evaluation is performed directly by the parents, they could refer mainly to family functioning considering that parent involvement in obsessive-compulsive symptoms is likely to contribute to impaired family relations because of the conflict due to the manifestation of symptoms. In addition, OCD children and adolescents in both groups (Low and High Accommodation) were without pharmacological and/or psychosocial treatment (nor individual child nor family-based treatment) at the time of evaluation. This reinforces our results. Finally, this is the first study conducted on OCD children and adolescents where the impact of the FA, in terms of psychological distress, has been studied on parents separately (mothers and fathers).

This study also has several limitations. First, within the total sample, specifically in the majority of participants, OCD symptoms were present in association with a primary diagnosis of Autism Spectrum Disorder, Attention Deficit/Hyperactivity Disorder, Tourette Disorder and Tic Disorder. Consequently, the final sample of children and adolescents with primary diagnosis of OCD was small. Secondly, the psychological distress of single parent was examined with a self-report questionnaire (SCL-90-R). To enhance the validity of our results on this aspect, an instrument based on the examiner’s clinical judgement could be used in future studies.

## Conclusion

In accordance with the level of global, social, and role impairment, and the high level of depressive symptoms that Family Accommodation causes on OCD children and adolescents and with the level of individual psychological distress that it induces in parents, it should be considered in the assessment and treatment of pediatric OCD. Indeed, OCD children, adolescents and their families should be supported, as part of a CBT approach, providing initial intervention addressed separately to the children or the adolescents and to their parents. For example, interventions aimed at reducing FA and parental psychological distress, if carried out immediately after the assessment and then in the initial phase of treatment, can increase the compliance of the parent to more structured protocols such as the exposure and prevention of response in which the parent is called on to support the child following the instructions of the psychotherapist, and the CBFT. Future and larger sample studies of children and adolescents with OCD and their parents however will need to replicate these results.

## Data Availability

The datasets used and/or analysed during the current study are available from the corresponding author on reasonable request.

## Abbreviations

CBT: Cognitive Behavioral Therapy
CBFT: Cognitive-Behavioral Family-based Treatment
CDI 2: Child Depression Inventory 2
CGAS: Childhood Global Assessment Scale
CY-BOCS: Children’s Yale-Brown Obsessive–Compulsive Scale
DSM-5: Diagnostic and Statistical Manual of Mental Disorders. 5th edition
FA: Family Accommodation
FAS: Family Accommodation Scale
GF: Role: Global Functioning: Role Scale
GF: Social: Global Functioning: Social Scale
GSI: Global Severity Index
IQ: Intelligence quotient
K-SADS-PL DSM-5: Schedule for Affective Disorders and Schizophrenia for School Aged Children Present and Lifetime Version DSM -5
MASC 2: Multidimensional Anxiety Scale for Children 2
OCD: Obsessive-Compulsive Disorder
PSDI: Positive Symptom Distress Index
PST: Positive Symptom Total
SCL 90-R: Symptom Checklist-90-R
WISC-IV: Wechsler Intelligence Scale for Children
